# The borderline between cluster headache and migraine: does cluster-migraine exist?

**DOI:** 10.1186/1129-2377-14-S1-P42

**Published:** 2013-02-21

**Authors:** C Lisotto, F Mainardi, F Maggioni, G Zanchin

**Affiliations:** 1 Headache Centre, University of Padua, Italy; 2 Headache Centre, Hospital of Venice, Italy

## Introduction

Cluster headache (CH) is a well definite disorder. When the attacks fulfil all but one of the criteria for CH, probable CH should be diagnosed, requiring one of the following conditions: 1) attacks lasting >180 minutes, 2) attacks without autonomic local signs or restlessness, 3) sporadic (less than one every other day) attacks. Background In the past “cluster-migraine” was considered an atypical variant of CH, but this entity was never categorized.

## Methods

Since 1996 we have observed 251 patients with CH. Out of these cases, 33 (19 males and 14 females) could not fulfil all criteria for CH and have been followed-up for at least 5 years.

## Results

We could distinguish 4 different subgroups. For 3 subgroups the unfulfilled criteria were: 1) pain duration >3 hours, ranging 4-8 hours (6 cases), 2) absence of autonomic signs or restlessness (5 cases), 3) sporadic attacks, with no cluster periodicity (10 cases). We could also identify a fourth subgroup of 12 patients without cluster pattern and attack duration lasting 3-5 hours, borderline between CH and migraine without aura (MO). Moreover, the coexistence of MO and CH was noted in 8 cases. The first subgroup overlaps with probable MO. Criteria are not fully met and patients are labelled as probable MO or probable CH, either of which could have features of the other. The second and third subgroups meet criteria for probable CH. The fourth subgroup does not fulfil criteria either for probable CH or probable MO, therefore the old definition of cluster-migraine may be still appropriate, even if this term might be considered a regression to the time when CH was considered a variant of migraine [1]. Interestingly, 3 patients in the third subgroup evolved over time into a typical CH.

## Conclusion

Patients sometimes present with clinical scenarios having characteristics of both MO and CH, but either do not fully meet criteria for either disorder or have no sufficient symptoms to allow both diagnoses to be present. These occasions may account for the controversial form of cluster-migraine.
